# Impact of Sulphate Ions Content on Performance of Maleic and Acrylic Superplasticizers in Cement Paste

**DOI:** 10.3390/ma14102683

**Published:** 2021-05-20

**Authors:** Elżbieta Janowska-Renkas

**Affiliations:** Department of Building Materials Engineering, Faculty of Civil Engineering, Opole University of Technology, Katowicka 48, 45-061 Opole, Poland; e.janowska-renkas@po.edu.pl

**Keywords:** cement, Calcium Sulphate (CaSO_4_, CaSO_4_·2H_2_O and CaSO_4_·0.5H_2_O), rheology, calorimetry, acrylic superplasticizer, maleic superplasticizer chemical structure SP, hydrophilicity of SP polymer

## Abstract

The paper presents test results of the impact of sulphate ions from calcium sulphates: Hemihydrate, dihydrate and anhydrite, on rheological properties and hydration heat of cement pastes with, and without, superplasticizers, derivatives of maleic (SP-2) and acrylic (SP-1) acids. It is demonstrated that cement pastes fluidity depends on superplasticizer chemical structure, and its effect is expressed by a hydrophilic coefficient. As maleic superplasticizers have flexible comb-like structure composed of a shorter backbone chain containing COO^−^ carboxylate groups and very long side chains, cement pastes showed higher fluidity than with acrylic superplasticizer with ladder-like structure, longer backbone chains with shorter side chains. SP-1 showing lower hydrophilicity coefficient and fewer COO^−^ groups was found to be less sensitive to increased sulphate ion content in pastes. However, with SP-2 with higher hydrophilicity, a gradual fluidity loss (increased paste viscosity) was observed. Plastic viscosity was approximately at the same level in SP-1-containing pastes. Tests showed that sulphates definitely changed polycarboxylate superplasticizers performance. A high concentration of sulphate ions reduced maleic superplasticizer efficiency. Under these conditions, SP-1 is more effective and therefore more suitable for fluidity of pastes containing higher SO_4_^2−^ ions content. Thus, sulphate ions concentration in the paste should be considered when selecting superplasticizer.

## 1. Introduction

Rheological properties of the cement paste depend on the amount and form of the calcium sulphate applied as a setting time regulator. The setting time regulator, when properly selected, assures effective regulation of C_3_A hydration process, which is demonstrated by the increased fluidity of the cement paste [1,2,3].

The introduction of superplasticizers to cementitious paste increases their fluidity and delays the cement hydration process suitably to the superplasticizer efficiency that depends on the performance mechanism. The performance mechanism of various types of superplasticizers has been described by numerous authors [4,5,6].

Currently applied new-generation superplasticizers are based on polycarboxylates (PC), copolymers of the acrylic acid with acrylates (CAE), as well as cross-linked acrylic resins (CLAP). They are built of a polyacrylic or polymethacrylic backbone chain containing side chains formed by carboxylate groups, polyoxyethylene, polyoxypropylene or polyether, ether-ester mers (units) or their mixtures [7,8,9]. Mechanism of their functioning, despite an electrostatic repulsive force of the same sign charges that are formed on the cement grain surface in result of superplasticizer adsorption, is associated with an effect of a spatial spherical blockade-created by long side poly(oxyethylene) chains present in the structure of these superplasticizers [10,11,12,13,14].

Efficiency of these superplasticizers may be affected by numerous factors depending on, besides others, their molecular structure, including; length and type of the backbone chain, length, number and density of side chains, presence of hydrophilic and hydrophobic groups in side chains, polymerisation degree and energy of adsorption bonds [13,15,16,17,18,19,20,21,22,23,24].

The interactions within cement-additive system—degree of the paste fluidity—are also determined by the superplasticizer form and its place within cement paste (organic-mineral phase, OMP), hydrated surface of cement grains or non-adsorbed form in the water phase [25].

The above was confirmed by research conducted by Uchikawa [26], which showed that the lower adsorption of the superplasticizer affected the growth of cement paste fluidity. Whereas, Jolicoeur et al. [27], proved the paste fluidity is proportional to the concentration of free (non-adsorbed) superplasticizer left in the cement paste solution.

According to former studies, the calcium sulphates added to the cement as the setting time regulator can react with superplasticizers, but these interactions may take different forms.

It should be emphasized that as both acceleration and delay, as well as no impact of the superplasticizer are noted in many papers, and in that, respect there is no clear answer.

The quantity and reactivity of C_3_A phase also have the influence on rheological properties of pastes with regard to the superplasticizer [28].

It results from the fact that calcium sulphates react with a tricalcium aluminate and depending on the content and type (hemihydrate or dihydrate) of the sulphate used, they delay hydration of the tricalcium aluminate to varying degrees, thus accelerating hydration of silicates [1,2,3]. Whereas, the quantity of gypsum required in the hydration process grows along with the content of C_3_A and alkalis [29,30]. A similar effect was found by authors of papers [31,32,33] for pastes in presence of the superplasticizer.

According to Roberts et al. [34], large concentration of the C_3_A phase and low accessibility of the sulphate ions in a paste, in presence of superplasticizer, deteriorates the rheological properties. On the other hand, large amount of C_3_A causes enhanced adsorption of superplasticizer on the C_3_S or C_2_S phase grains, thus, improving pastes rheological properties.

Whereas, according to Pourchet et al. [25], low reactivity of the C_3_A phase during the first hydration period leads to deterioration of rheological properties of pastes. This is caused by building the superplasticizer molecules into the products of aluminates hydration and consequently by decrease of the polymer amount available to dispergate the cement particles agglomerates.

Many authors, including Grierson et al. [28], Jolicoeur et al. [35], Sakai et al. [36] found that the superplasticizer may block reactions of sulphate ions coming from gypsum with C_3_A phase, which in consequence led to a competitive reaction of the superplasticizer with the tricalcium aluminate. The competitive adsorption of sulphate ions on the surface of hydrated C_3_A [37,38] causes (in the first 30 min. of the hydration period) that a larger quantity of the superplasticizer is built into the organic-mineral phase (OMP) at the small quantity of SO_4_^2−^ ions. It causes a reduced quantity of the superplasticizer required for effective dispersion of cement particles. On the other hand, if the sulphate ions quantity is sufficient, a larger quantity of superplasticizer polymer particles causes enhanced fluidity of cement pastes [24].

This is also confirmed by the results obtained by Hanna et al. [39] who proved that at the small calcium sulphate dissolution rate, superplasticizer molecules tend to adsorb on aluminates, thus preventing ettringite formation reaction. To prevent superplasticizer molecules interference into the ettringite formation process, the authors of this paper are of the opinion that better accessibility of SO_4_^2−^ ions in the solution is necessary during hydration. Therefore, the suitable calcium sulphate dissolution rate, adjusted to C_3_A phase reactivity, is so important. Furthermore, the dissolution rate of calcium sulphates becomes lower in the presence of sulphate ions from superplasticizer function groups.

Whereas, according to research conducted by Nakajama and Yamada et al. [37], along with the growth of sulphate ions in the solution, the quantity of superplasticizer molecules adsorbed on the cement grains is reduced, leading to lower fluidity of cement pastes. It is also confirmed by Pourched et al. [25].

According to the above review, influence of the sulphate ions (originated from the sulphates displaying different dissolution rate) on efficiency of the new-generation superplasticizers is still not clear despite intensive researches.

Due to diverse opinion, this study tries to explain the relation between sulphate ions concentration (which depends, in turn, on the dissolution rate of sulphates applied) and the efficiency of the new-generation superplasticizers that base on polycarboxylic ethers of various chemical and molecular structure.

## 2. Materials and Methods

Material used for testing was cement prepared in a lab by grinding the industrial clinker together with the setting time regulator.

Chemical and mineral composition of the clinker and the specific surface area are shown in Table 1. The specific surface area of clinker was determined acc. to Blaine and was 314.0 m^2^/kg.

As the setting time regulator, various forms of calcium sulphates were used (CaSO_4_, CaSO_4_·2H_2_O and CaSO_4_·0.5H_2_O) and additionally mixtures of CaSO_4_ and CaSO_4_·0.5H_2_O sulphates in amount of 5% by mass.

The quantity of CaSO_4_ in the mixture fluctuated from 20 to 80% by mass. In all cements made the total quantity of sulphates introduced into cements was the same and it amounted to 2.33% SO_3_ (Table 2).

Introduction of various forms of calcium sulphates with different dissolution rates to the clinker as the setting regulator, (Table 3) and their various quantity aimed at differentiaion of sulphate ions quantity in the solution at the initial stage of cement hydration.

As it is known [40], the quantity of sulphate ions in the solution increases along with the growth of the calcium sulphate dissolution rate. SO_4_^2−^ ions, bound in the calcium sulphate hemihydrate, penetrate the solution as first, then ions in the calcium sulphate dihydrate, and at last ions of natural anhydrite. The above correlation is also confirmed by results of testing obtained in this paper (Table 3).

Two types of new-generation technical superplasticizers (SP), polyoxyethylene derivatives of carboxylic acids (SP-1 and SP-2) were used for testing. SP-1 and SP-2 superplasticizers were applied as 40% solutions and constituted 1 wt% of the cement mass.

## 3. Testing Methods

### 3.1. Gel Permeation Chromatography (GPC)

In order to determine a phase composition of superplasticizer test specimens, they were subject to chromatographic distribution into polymer fractions using a method of gel permeation chromatography (GPC) with the use of GPC chromatograph Watt Technology with RI refraction detector and Down EOS—a multi-angle light scattering detector. THF (tetrahydrofuran) was used as an eluent. The chromatographic distribution of superplasticizers in a form of solutions with concentration 1–2% was performed on GPC column in temp. 35 °C at eluent flowrate THF 1 mL/min with application of a set of gel columns PSS SDV 1·10-6A+2·100A size 300 mm × 8 mm (Polymer Standard Service).

### 3.2. Fourier Transform Infrared Spectroscopy (FTIR)

Superplasticizers testing by means of IR absorption spectroscopy was conducted using a FTIR spectrometer—PU 9804 model of Philips Analytical (UK), while keeping constant resolution of spectrum equal to 2 cm^−1^ after twenty scans of each KBr pellet containing the specimen tested. Recording was performed in a full spectrum range 4000–400 cm^−1^ using EAGLE v.4.31 computer software.

To determine the content of hydrophobic aliphatic groups: CH, CH_2_, ester groups: O=C-O-CH_2_ and hydrophilic polyoxyethylene groups: O-CH_2_CH_2_, IR absorption spectra (FTIR) were made for superplasticizers in a solid state. Spectra were standardized to the equal content of specimens in the spectrum beam by means of aliphatic group band, i.e., 2887 cm^−1^ STRCH, CH_2_.

### 3.3. Rheological Testing

Rheological measurements of pastes were conducted using Viscotester VT550 rotational HAAKE viscometer, Karlsruhe, Germany with concentric cylinders. The plastic viscosity of pastes was determined based on the flow curve set for shear rates going up and down within the range from 0 to 150 s^−1^. The yield stress and plastic viscosity values were calculated based on a Bingham model. Measurements were conducted for cement pastes containing superplasticizer in amount of 1% by mass, while keeping the equal water-to-solid phase ratio (*w*/*s*) equal to 0.39 and the constant temperature 21 °C.

### 3.4. Hydration Heat Testing

A microcalorimeter of JAF manufactured by Wexham Developments, Reading, UK was used to test hydration heat of cements. Tests were conducted on test specimens of the cement paste with addition of 1% by mass of superplasticizer during 48 h, while keeping a constant w/c ratio equal to 0.4 and the constant temperature 21 °C.

## 4. Results of Testing and their Interpretation

### 4.1. Test Results of Superplasticizers Structure

**Figure 1:**
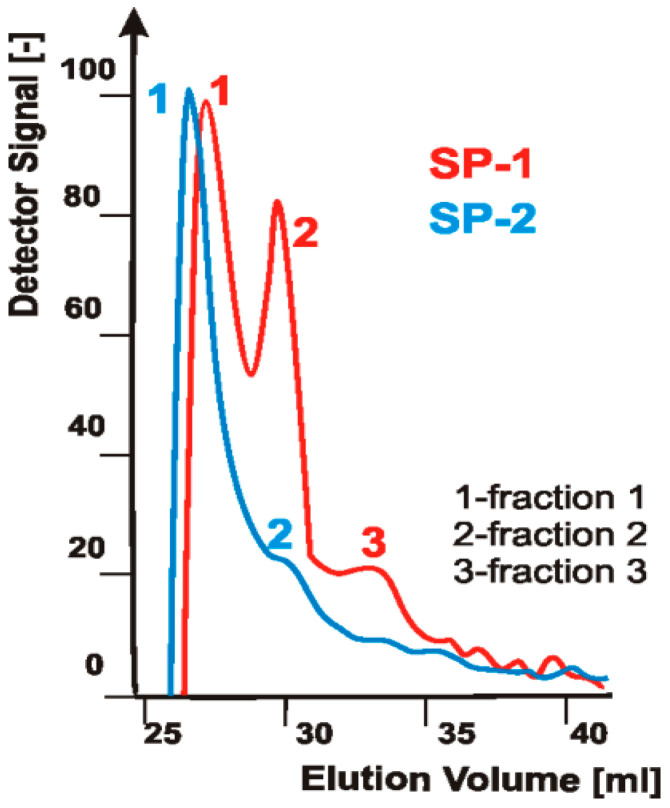
GPC chromatograms of superplasticizers specimens (SP-1, SP-2), [based on results published in [41]].

Based on GPC tests (Figure 1), presence of two different polymer fractions was found in SP-2 specimen, whereas in SP-1 specimen the additional third fraction was found. Polymer fractions 1 originated from pure superplasticizers, while fractions 2 and 3 from a part of unreacted poly(ethylene glycols) (PEG) used in the synthesis of superplasticizers.

**Figure 2:**
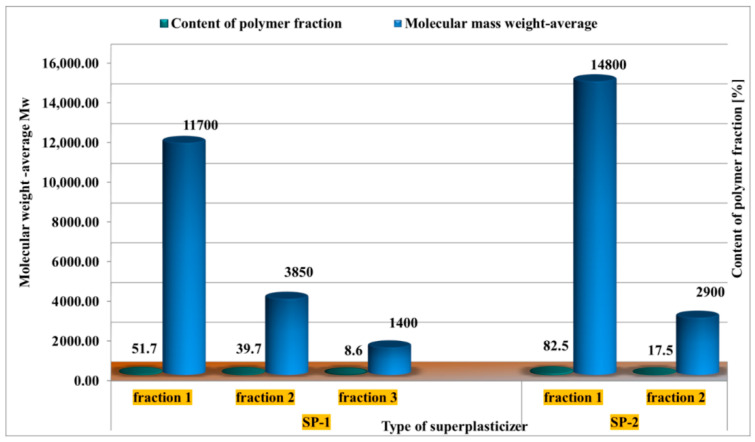
Results of the GPC chromatographic analysis of superplasticizer polymer fractions (SP-1, SP-2), [based on results published in [41]].

Individual polymer fractions present in superplasticizers specimens differed with each other with a molar mass and polydispersity (MWD = Mw/ML molecular weight distribution (Figure 2).

Molecular mass weight-average (Mw) of SP-1 superplasticizer was ca. 12,000 g/mol, and as shown by GPC testing, it was heavily contaminated with residual poly(ethylene glycols), content of which was 48.3%. Whereas, SP-2 specimen had higher molecular mass weight-average equal to ca. 14,000 g/mol and much lower content of free poly(ethylene glycols), i.e., 14–18%. Therefore, it contained more pure polymer of the superplasticizer (ca. 82%), which for SP-1 specimen amounted to 51%.

**Figure 3:**
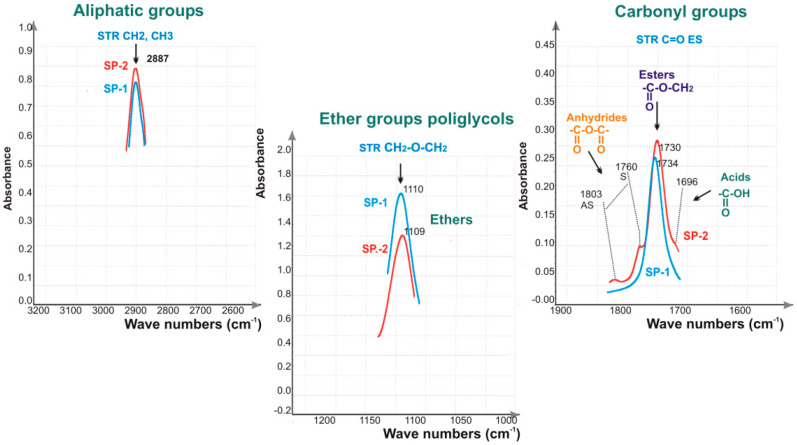
Selected function groups: Aliphatic, ether and carbonyl groups identified by means of IR testing), (based on results published in [41]).

GPC testing showed that superplasticizers tested were polyoxyethylene derivatives of carboxylic acids. SP-1 specimen originated from esterification of acrylic acid oligomers with a mixture of poly(ethylene glycols) PEG 1500 + PEG 4000. Whereas SP-2 specimen originated from esterification of maleic anhydride oligomers by means of poly(ethylene glycol) PEG 3000. Approximate structures of superplasticizers are presented in Figure 3.

**Figure 4:**
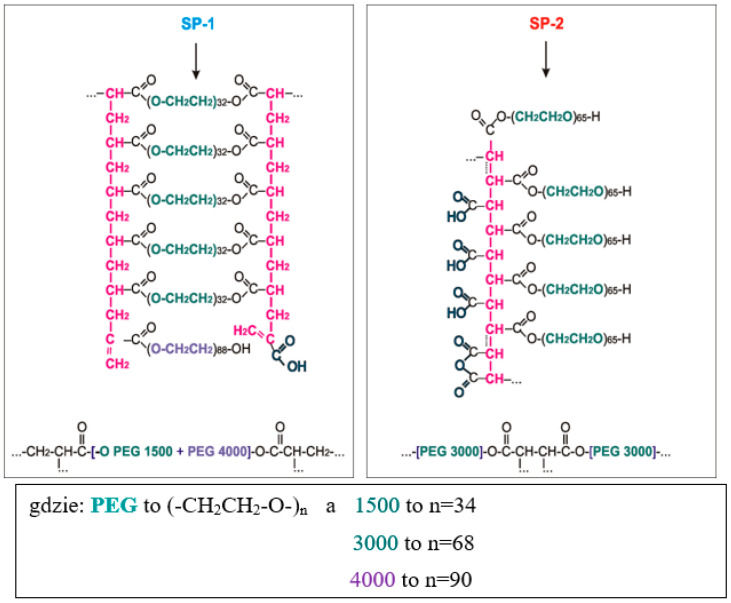
Approximate chemical structures of superplasticizers: ladder-shaped (SP-1) and comb-shaped (SP-2) determined based on mathematical calculation of molar masses.

Based on FTIR spectra, presence of characteristic function groups was found in superplasticizers in selected spectrum bands for aliphatic groups frequency (3200–2500 cm^−1^), carbonyl groups (1900–1500 cm^−1^) and ether groups (1250–950 cm^−1^) (Figure 4).

FTIR spectra characteristic for carbonyl groups 1500–1900 cm^−1^ for SP-2 specimen, apart from esters (1730–1734 cm^−1^), certain amounts of free carbonyl acids were found (1696 cm^−1^), as well as the maleic anhydride (doublet 1803, 1760 cm^−1^).

**Figure 5:**
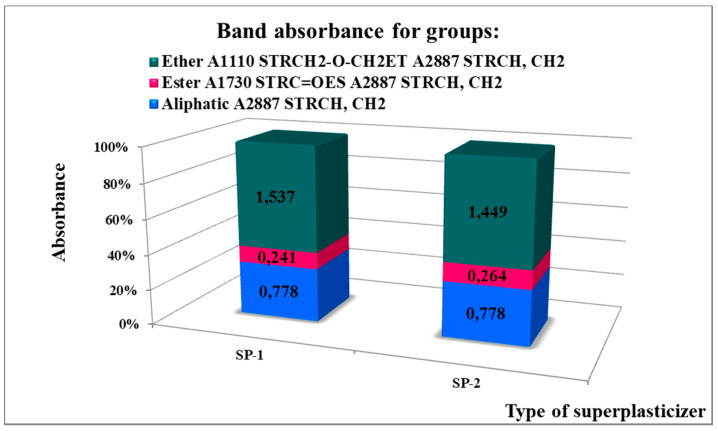
Results of FTIR spectral analysis of superplasticizer samples (based on results published in [41]).

Absorbance values of bands specific for hydrophobic aliphatic groups read from FTIR spectra (Figure 4) with the wave number 2887 cm^−1^ STRCH, CH_2_ and ester groups with the wave number 1730–1734 cm^−1^ STRC = OES, and for hydrophilic oxyethylene groups 1109–1112 cm^−1^ STRCH_2_-O-CH_2_ET for the poly (ethylene glycol) chains of free PEGs, as well as for the ester groups present in superplasticizers are presented in Figure 5.

Based on polymer fraction weight content determined with the GPC method for SP superplasticizer (fraction 1) and free PEG (fraction 2, and possibly fraction 3) in specimens tested, distribution (content) of ether groups absorbance in both polymers was defined (Table 4).

Based on FTIR test results, hydrophilicity of superplasticizers was calculated (Table 4) following a definition given by Grzeszczyk and Sudoła [42].

It was found that SP-2 superplasticizer had the higher hydrophilicity (a ratio of absorbance of hydrophilic ether groups AET^1110^ to hydrophobic ester groups AES^1730^) equal to A_ET_^1110^/A_ES_^1730^ = 4.53, compared to hydrophilicity of SP-1 superplasticizer, i.e., A_ET_^1110^/A_ES_^1730^ = 3.30, which suggest high probability of SP-2 better efficiency in hydrophilic solvents.

### 4.2. Results of Rheological Testing

**Figure 6:**
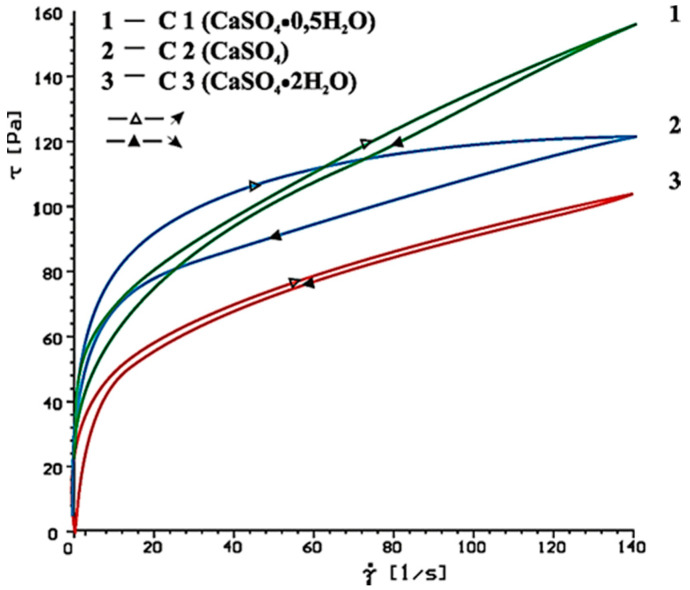
Flow curves of cement pastes with addition of CaSO_4_·0.5H_2_O, CaSO_4_, CaSO_4_·2H_2_O, after 10 min.

**Figure 7:**
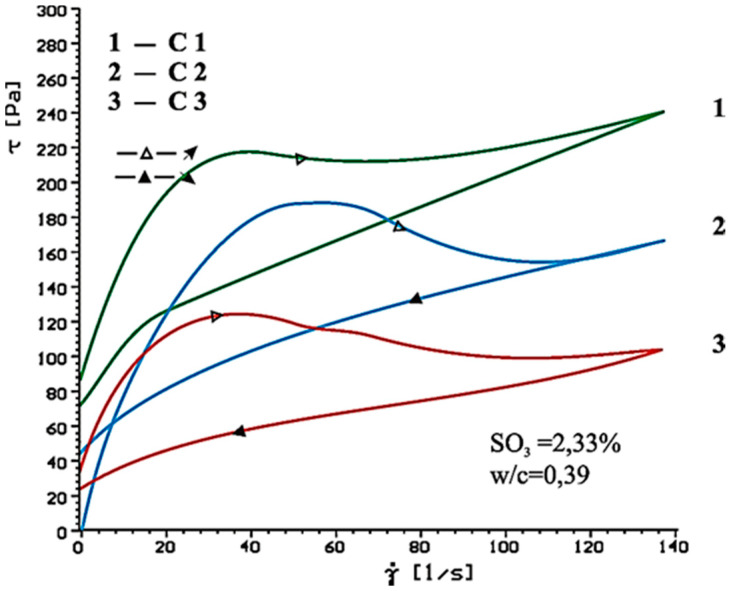
Flow curves of cement pastes with addition of CaSO_4_·0.5H_2_O, CaSO_4_, CaSO_4_·2H_2_O, after 60 min.

**Figure 8:**
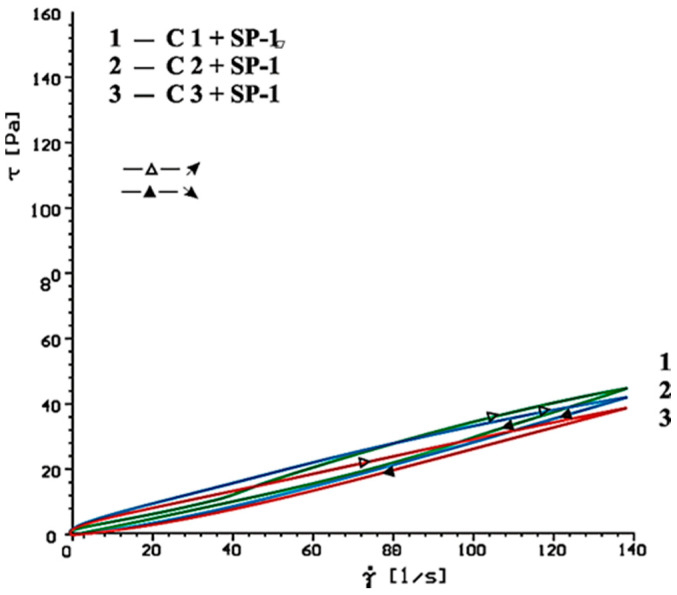
Flow curves of cement pastes with addition of CaSO_4_·0.5H_2_O, CaSO_4,_ CaSO_4_·2H_2_O in presence of SP-1 superplasticizer, after 10 min.

**Figure 9:**
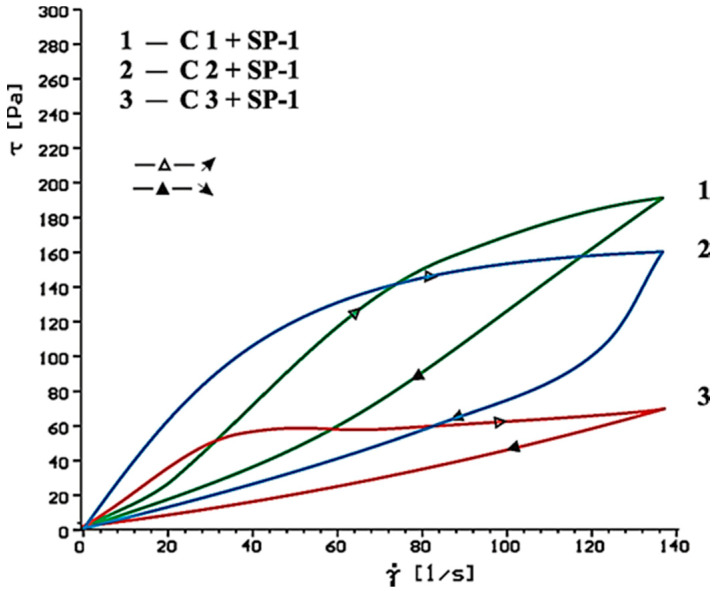
Flow curves of cement pastes with addition of CaSO_4_·0.5H_2_O, CaSO_4_, CaSO_4_·2H_2_O in presence of SP-1 superplasticizer, after 60 min.

**Figure 10:**
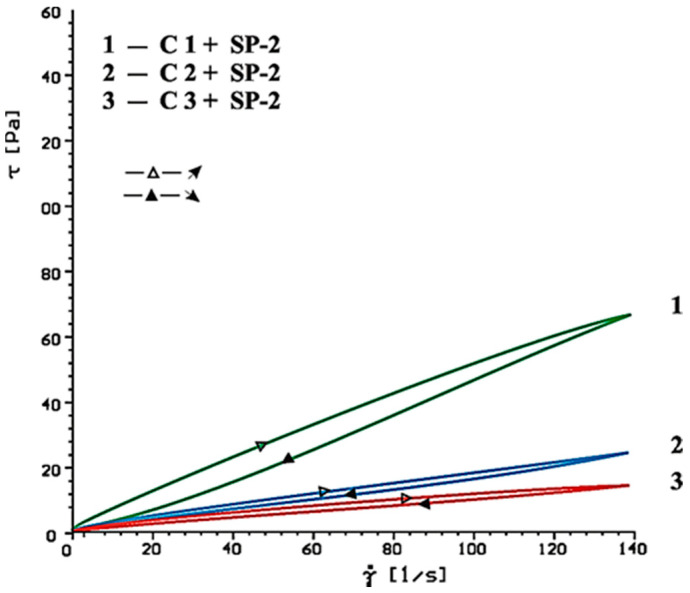
Flow curves of cement pastes with addition of CaSO_4_·0.5H_2_O, CaSO_4_, CaSO_4_·2H_2_O in presence of SP-2 superplasticizer, after 10 min.

**Figure 11:**
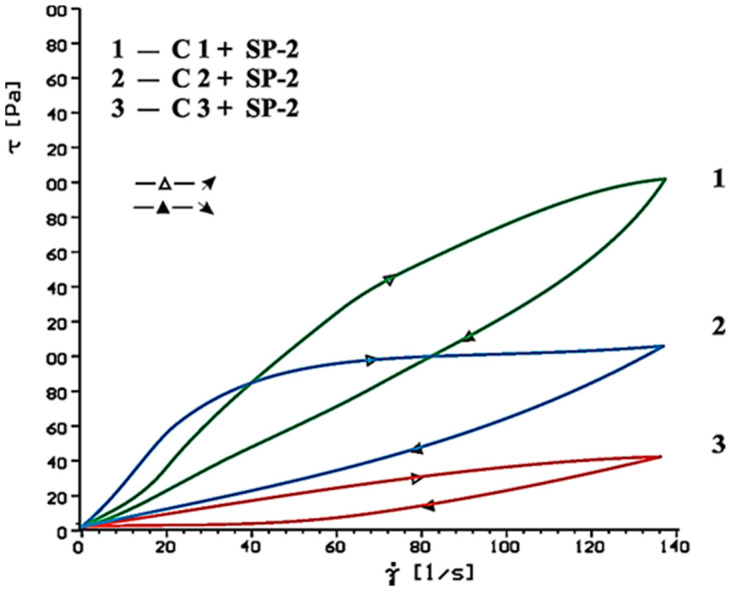
Flow curves of cement pastes with addition of CaSO_4_·0.5H_2_O, CaSO_4_, CaSO_4_·2H_2_O in presence of SP-2 superplasticizer, after 60 min.

Flow curves of cement-based pastes, containing calcium sulphates (CaSO_4_, CaSO_4_·2H_2_O, CaSO_4_·0.5H_2_O) as the setting time regulator, without and with SP-1 and SP-2 superplasticizers after 10 and 60 min of hydration are presented in Figure 6, Figure 7, Figure 8, Figure 9, Figure 10 and Figure 11.

The Table 5 shows yield stress and plastic viscosity values for cement pastes tested.

The addition of various forms of calcium sulphate (with various dissolution rates) to the cement as the setting time regulator, aimed at differentiation of sulphate ions concentration in the solution at the initial stage of cement hydration.

Calcium sulphates used had different dissolution rates (Table 3), thus, accessibility of sulphate ions in the paste was diverse. The quantity of sulphate ions in the solution grows along with the increase of the dissolution rate of individual types of the calcium sulphate [43]. SO_4_^2−^ ions, originating from the calcium sulphate hemihydrate, penetrate the solution as first, then ions from the calcium sulphate anhydrite, and at last ions of calcium sulphate dihydrate.

In the analysis of results the following identification was introduced for cements containing various forms of calcium sulphate: C1 contains CaSO_4_·0.5H_2_O, C2-CaSO_4_ and C3-CaSO_4_·2H_2_O (Table 3). The total quantity of sulphates introduced into cements was the same and it amounted to 2.33% SO_3_.

Flow curves of cementitious pastes without and with 1% by mass content of SP-1 acrylic superplasticizer or SP-2 maleic superplasticizer after 10 and 60 min of hydration are presented in Figure 6, Figure 7, Figure 8, Figure 9, Figure 10 and Figure 11, Table 5 shows yield stress and plastic viscosity values for pastes tested.

As could be expected, the highest yield stress and plastic viscosity value was demonstrated by a cementitious paste (C1) with a gypsum hemihydrate. The plastic viscosity of this paste was twofold higher than the plastic viscosity of the paste containing CaSO_4_ (C2) and CaSO_4_·2H_2_O (C3). It is caused by false binding (formation of gypsum crystals) in case of solution supersaturation in the cement paste is compared to gypsum [3].

Much lower value of rheological parameters for C2 and C3 pastes is a result of delayed hydration process of C_3_A phase with addition of gypsum dihydrate and anhydride. However, cementitious pastes containing anhydrite as the setting time regulator showed higher yield stress and plastic viscosity than pastes with calcium sulphate dihydrate. The following relation was observed by Bensted [40] and Bundyra-Oracz [44].

Based on obtained results of rheological tests of pastes containing different forms of calcium sulphates, better efficiency was found for SP-2 superplasticizer compared to SP-1. Undoubtedly it results from different chemical and molecular structure of the superplasticizers used, particularly amount of hydrophilic groups determined by a coefficient of hydrophilicity.

The SP-2 superplasticizer that has a higher hydrophilicity coefficient (AET^1110^/AES^1730^ = 4.53), built from shorter backbone chains, containing more COO^−^ carboxylate groups, and very long hydrophilic polyether side chains (n = 68 oxyethylene mers in diols, PGE 3000, Figure 3), shows better efficiency compared to SP-1 superplasticizer with a lower hydrophilicity coefficient (AET^1110^/AES^1730^ = 3.30). That last one contains a longer backbone–methacrylic chain (with a smaller amount of carboxylate groups), to which shorter side chains are connected (n = 34 i n = 90 oxyethylene mers in chains of diols, PGE 1500 and PGE 4000, Figure 2).

The above conclusion can be generalized, i.e., the higher efficiency of the SP-2 superplasticizer results from long side chains and the COO^−^ groups (confirmed by the FTIR results) that make the cement flocculation difficult [14,17].

In case of higher concentration of SO_4_^2−^ ions after the shorter hydration time in C1 cement paste with addition of CaSO_4_·0.5H_2_O, with the highest dissolution rate (Table 3), efficiency of SP-2 superplasticizer is lower (η_pl_ = 0.51 Pa·s) than SP-1 superplasticizer (η_pl_ = 0.38 Pa∙s, Table 5). Under these conditions, the maleic superplasticizer (SP-2) does not inhibit formation of gypsum dihydrate in the cement paste.

In this case, the lower efficiency of the maleic superplasticizer (SP-2) (higher viscosity of the paste) may be explained by an impact of high sulphate ions concentration in the paste on impediment of COO^−^ carboxylate group adsorption on grains of C_3_A phase. The effect of sulphate ions that impede adsorption of carboxylate admixtures on cement grains, along with their increased content in the paste, was also demonstrated by Nakajima and Yamada [37] Aïtcin [45], and Zingg [46].

In case of SP-1 superplasticizer, due to a very small amount of COO^−^ groups, adsorption may occur through partial negative charges present in the backbone chain, formed in result of polarity of bonds in ester groups [47]. Polyether side chains, including in the superplasticizer, connected with the backbone chain simultaneously by two ester bonds, increase the distance between cement grains, and deflocculating them effectively. To some extent, it may explain the higher efficiency of the acrylic superplasticizer with the increased amount of sulphate ions.

It is worth to emphasize that with addition of SP-2 superplasticizer to the cement paste, in the initial period (10 min.), the influence of sulphate ions concentration in the paste (type of sulphate) is observed on its efficiency, unlike SP-1 superplasticizer, where no such effect is observed (Figure 8 and Figure 10). It is confirmed by the results of rheological testing of C1, C2 and C3 cement pastes that show minimum differences in plastic viscosity of pastes containing SP-1 superplasticizer. It proves a lower impact of sulphate ions content in the solution on paste fluidity by the acrylic superplasticizer. After 60 min, in both cases (SP-1 and SP-2), the impact of sulphate ions concentration on the increase of paste viscosity (higher calcium sulphate dissolution rate increases the paste viscosity) is visible, Figure 9 and Figure 11, Table 5). The highest plastic viscosity of the paste containing CaSO_4_·0.5H_2_O is most likely caused by a false bond.

**Figure 12:**
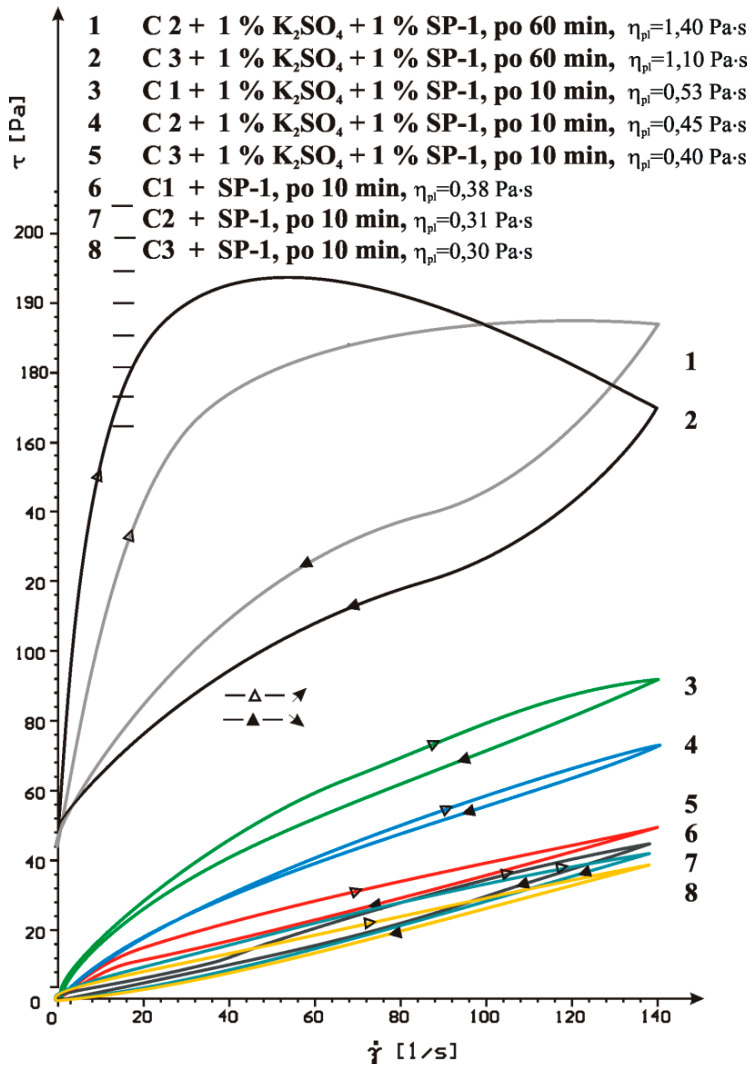
Flow curves of pastes of C1, C2 and C3 cements containing K_2_SO_4_ in presence of SP-1 in 1% by mass, after 10 and 60 min.

Effect of sulphate ions (SO_4_^2−^) on efficiency of SP-1 superplasticizer (less sensitive to sulphate content) was tested by introducing to cements C1, C2 and C3 (Table 2) the potassium sulphate with a significant dissolution rate, in the amount of 1% by mass (Figure 12).

The results of rheological testing showed that the introduction of the potassium sulphate caused a loss of fluidity in all pastes, i.e., C1, C2 and C3 already after 10 min. Whereas, after 60 min, the increase of rheological parameters was so big that the measurement in case of C1 cement paste containing gypsum hemihydrate was impossible. Flow curves of C2 and C3 cement pastes containing K_2_SO_4_ showed a clearly marked thixotropy with a large surface area of the hysteresis loop (Figure 12).

The significant increase of the yield stress and plastic viscosity of pastes observed may be caused by formation of a syngenite, which is reported by Kurdowski [48]. The increase of rheological parameters in pastes with K_2_SO_4_ was also observed by Andersen et al. [49].

**Figure 13:**
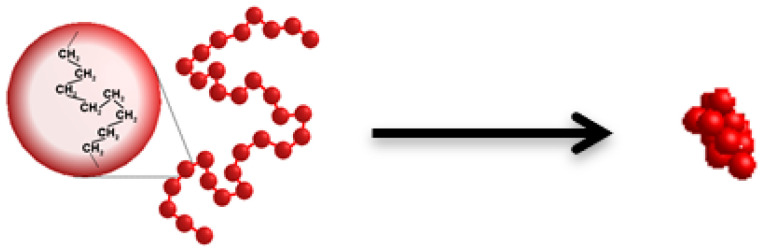
Two states of polymer in the solution: a freely unfolded chain and chain condensed under the stimulus impact-attraction of chain segments based on [52].

The introduction of the potassium sulphate (K_2_SO_4_) to the paste has affects a significant increase of ionic strength, which in the case of the superplasticizer containing polyoxyethylene chains (PEG), will lead to quick clustering of these side chains and reduction of steric effect (Figure 13) [50,51]. It may also affect the loss of paste fluidity (Figure 12).

**Figure 14:**
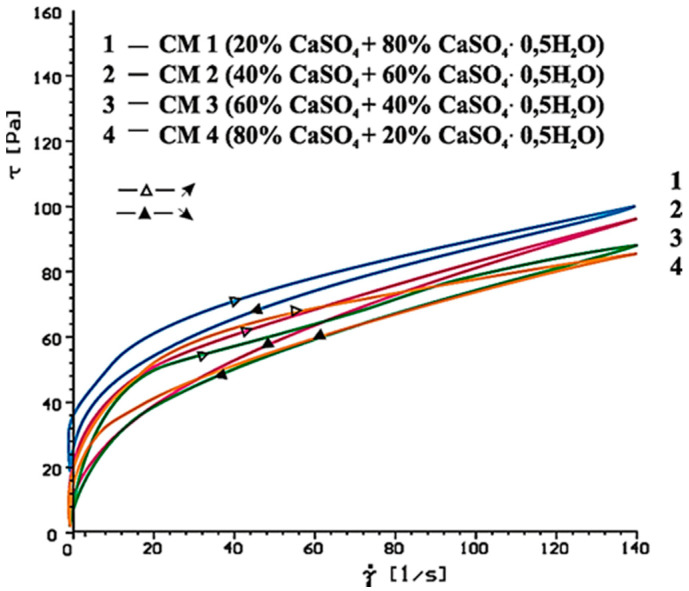
Flow curves of cementitious pastes containing 5% by mass of CaSO_4_·0.5H_2_O and CaSO_4_ in quantity from 20 to 80% by mass, after 10 min.

**Figure 15:**
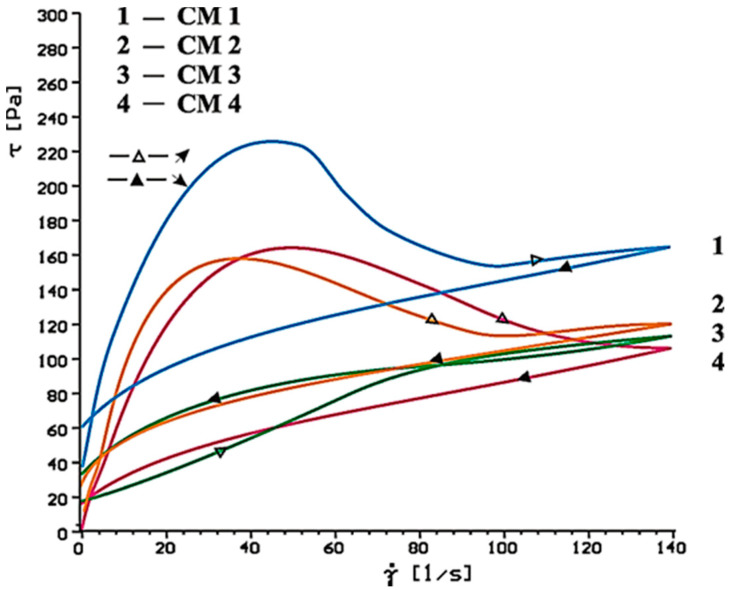
Flow curves of cementitious pastes containing 5% by mass of CaSO_4_·0.5H_2_O and CaSO_4_ in quantity from 20 to 80% by mass, after 60 min.

**Figure 16:**
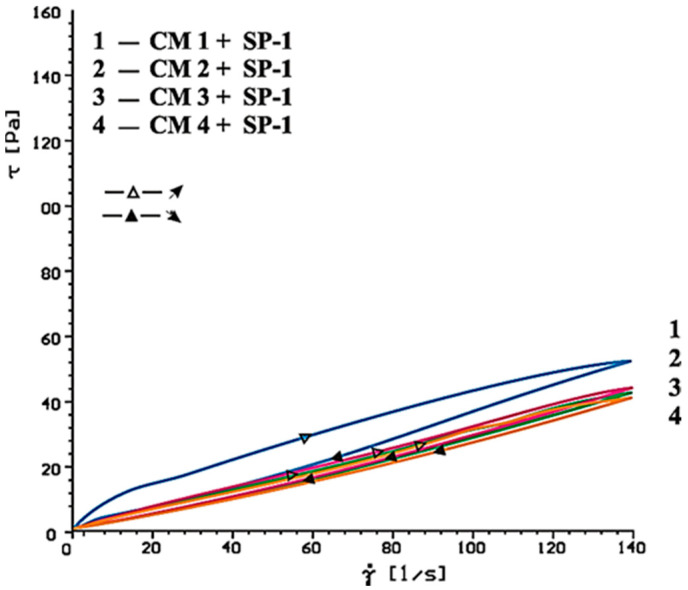
Flow curves of cementitious pastes containing 5% by mass of CaSO_4_·0.5H_2_O and CaSO_4_ in quantity from 20 to 80% by mass in presence of SP-1 type superplasticizer, 1% by mass, after 10 min.

**Figure 17:**
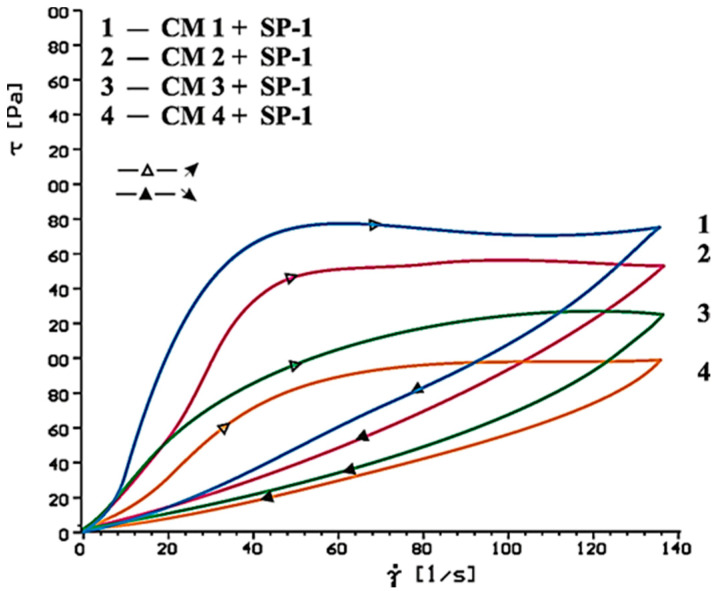
Flow curves of cement pastes containing 5% by mass of CaSO_4_·0.5H_2_O and CaSO_4_ in quantity from 20 to 80% by mass in presence of SP-1 type superplasticizer, 1% by mass, after 60 min.

**Figure 18:**
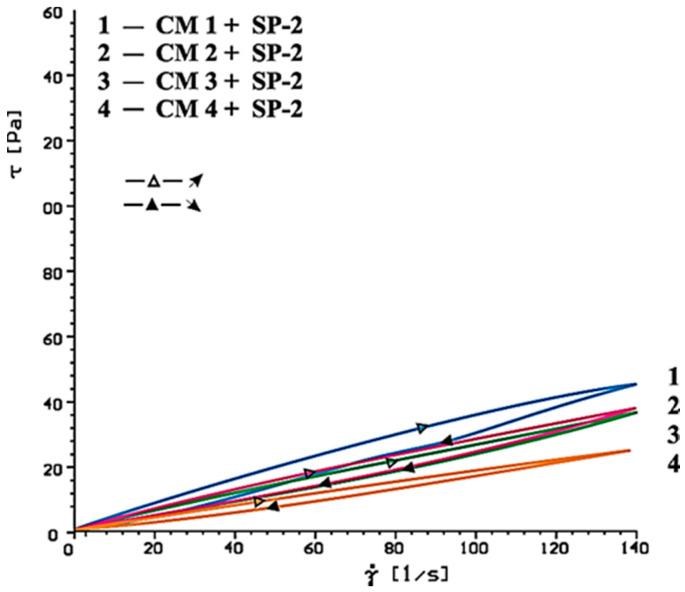
Flow curves of cementitious pastes containing 5% by mass of CaSO_4_·0.5H_2_O and CaSO_4_ in quantity from 20 to 80% by mass in presence of SP-2 type superplasticizer, 1% by mass, after 10 min.

**Figure 19:**
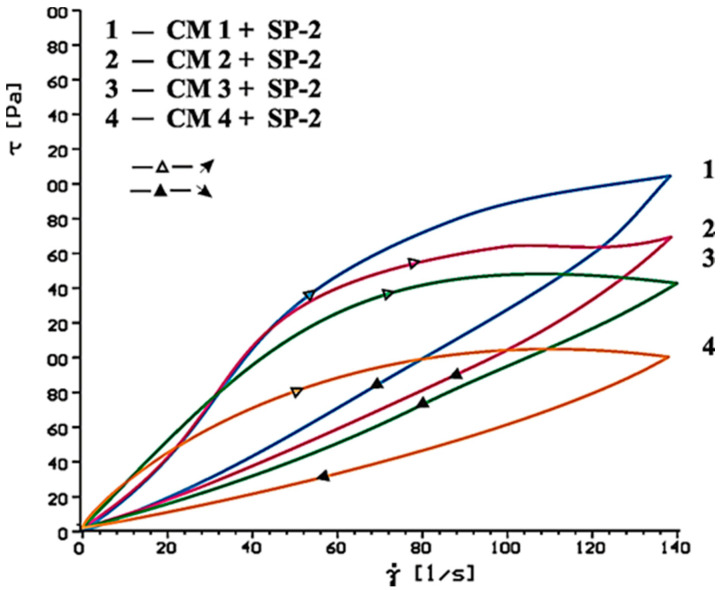
Flow curves of cement pastes containing 5% by mass of CaSO_4_·0.5H_2_O and CaSO_4_ in quantity from 20 to 80% by mass in presence of SP-2 type superplasticizer, 1% by mass, after 60 min.

Figure 14, Figure 15, Figure 16, Figure 17, Figure 18 and Figure 19 show flow curves of pastes made from cements (CM1, CM2, CM3 and CM4) containing mixtures of calcium sulphates CaSO_4_·0.5H_2_O and CaSO_4_ with, and without, SP-1 and SP-2 superplasticizers in amount of 1% by mass. The yield stress and plastic viscosity values determined for these pastes containing mixtures of sulphates are given in Table 6.

Flow curves of pastes made of cements containing mixtures of sulphates (CM1-CM4) z with SP-1 and SP-2 superplasticizers are presented in Figure 16, Figure 17, Figure 18 and Figure 19. The yield stress and plastic viscosity values determined for the pastes containing mixtures of sulphates are given in Table 6. The yield stress and plastic viscosity values determined for the pastes containing mixtures of sulphates are given in Table 6.

The results of rheological testing of pastes made of cements containing mixtures of calcium sulphate anhydrite and hemihydrate-with different dissolution rate value (composition of cements are presented in Table 3 show that gradual increase of calcium sulphate hemihydrate content in the mixture with the anhydrite (from 20% to 80% by mass) in CM4, CM3, CM2 and CM1 cements, cause the increase of rheological parameters of pastes from these cements (Figure 14 and Figure 15, Table 6). Therefore, the increase of SO_4_^2−^ sulphate ions concentration leads to gradual reduction of pastes fluidity.

In the presence of SP-1 superplasticizer, fluidity of pastes CM2, CM3, CM4 in the initial period (after 10 min) remains on a similar level, except CM1 pastes, where a significant content of CaSO_4_·0.5H_2_O may cause formation of the dihydrate, so the increase of the plastic viscosity is observed (Figure 16).

**Figure 20:**
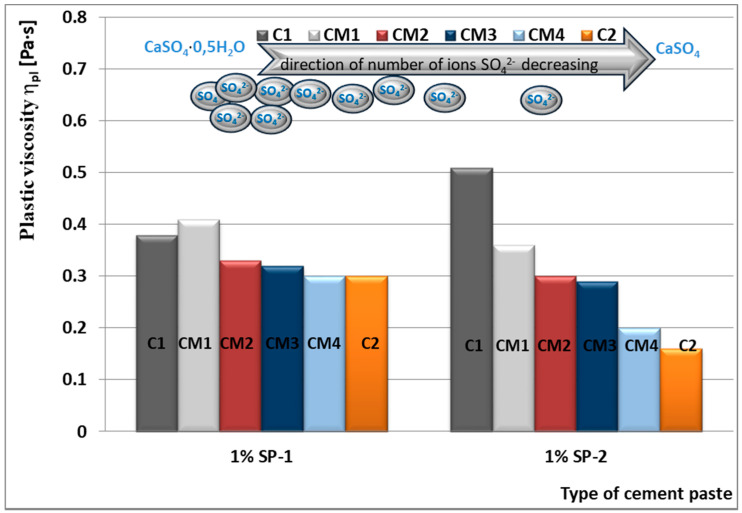
Plastic viscosity η_pl_ of pastes from cements C1, CM1, CM2, CM3 and C2 containing calcium sulphate anhydrite without and with 1% by mass of SP-1 and SP-2 superplasticizer, determined after 10 min.

Plastic viscosity values of CM2, CM3, CM4 pastes are comparable but higher than for analogous pastes containing SP-2 (Figure 20, Table 6).

A different situation is observed in the paste containing SP-2 superplasticizer. The increase of SO_4_^2^ ion concentration by introduction of more and more calcium sulphate hemihydrate to the mixture with the anhydrite, efficiency of this superplasticizer in the initial time (10 min.) is gradually reduced, which is demonstrated by the higher and higher plastic viscosity value of these pastes (Figure 18 and Figure 20, Table 6).

The above test results confirm lower sensitivity of the acrylic superplasticizer to quantity of sulphates in the cement paste (in the initial period), demonstrated earlier for pastes of cements C1, C2 and C3. After longer time (60 min) the increase of paste’s plastic viscosity is observed, in presence of both SP-1 and SP-2. In case SP-2 superplasticizer is used (as after 10 min), the viscosity increases along with the increase of SO_4_^2−^ ions concentration in the paste, and thus the content of hemihydrate gypsum in the mixture with anhydrite is higher (Figure 17 and Figure 19, Table 6).

Results of rheological testing of pastes containing various forms and different quantities of calcium sulphates (CaSO_4_·0.5 H_2_O and CaSO_4_) explicitly indicate that gradual increase of sulphate ions concentration in the paste causes gradual reduction of maleic superplasticizer (SP-2) efficiency, in contrast with the acrylic superplasticizer (SP-1). In case of SP-1, the increase of sulphate ions concentration in the paste in the initial period (10 min.) has practically no impact on its efficiency, except the paste containing a significant amount of sulphate ions, where the false bond occurrence is possible (Figure 20).

The test results indicate that SP-2 superplasticizer, which shows the highest hydrophilicity and the highest efficiency, in pastes with the proper control of the setting process (with good fluidity and addition of the setting time regulator), in case the content of SO_4_^2−^ ions in the paste grows, it reduces its efficiency.

The results of rheological tests of the above-mentioned pastes are analogous to results of hydration heat tests.

**Figure 21:**
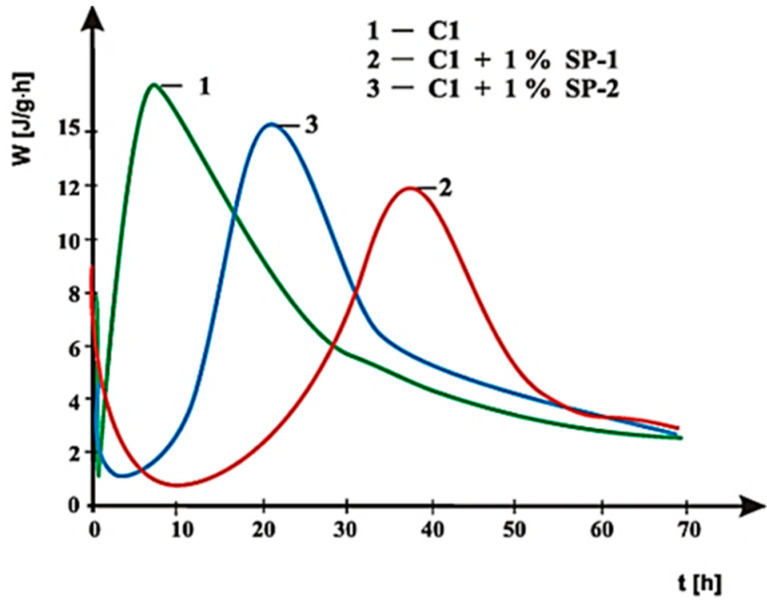
Hydration heat evolution rate curves for C1 cement without and with 1% by mass of SP-1 and SP-2 superplasticizers.

**Figure 22:**
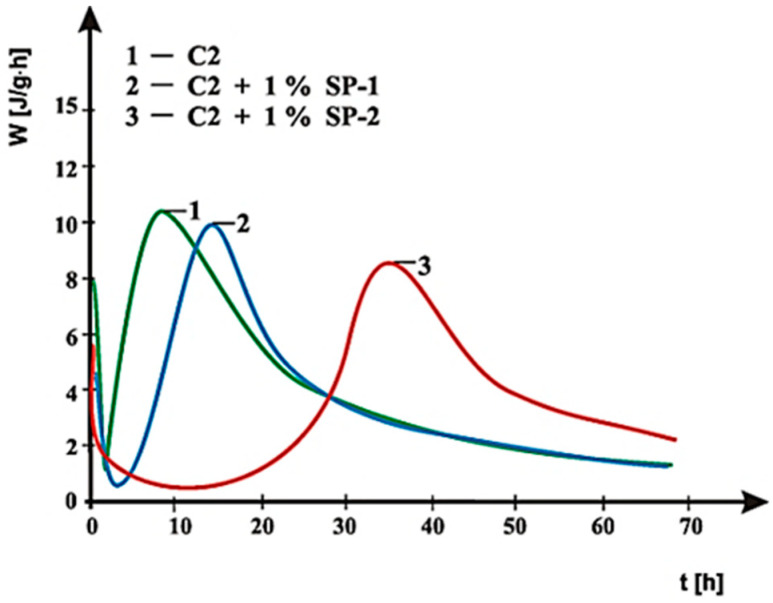
Hydration heat evolution rate curves for C2 cement without and with 1% by mass of SP-1 and SP-2 superplasticizers.

**Figure 23:**
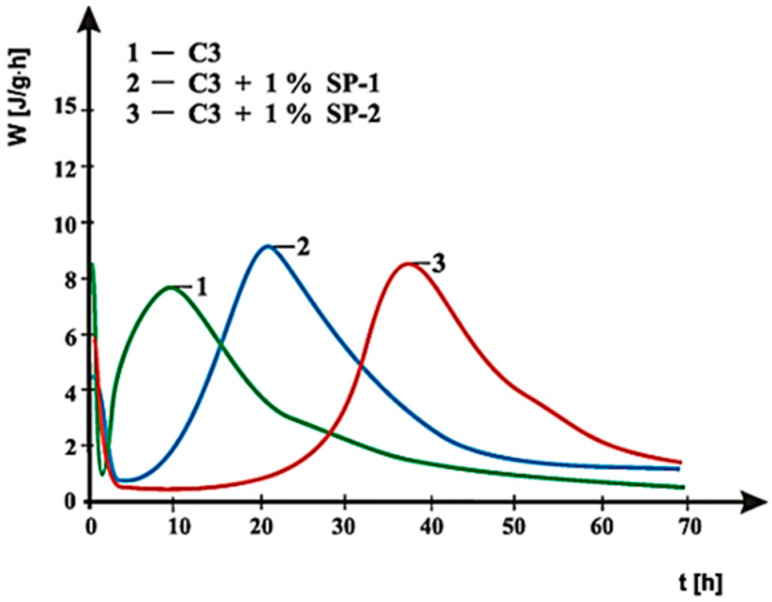
Hydration heat evolution rate curves for C3 cement without and with 1% by mass of SP-1 and SP-2 superplasticizers.

**Figure 24:**
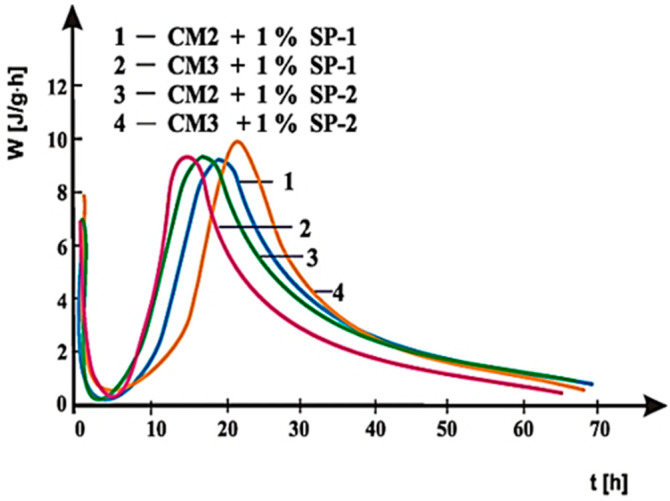
Hydration heat evolution rate curves for CM2 and CM3 cements with 1% by mass of SP-1 and SP-2 superplasticizers.

**Figure 25:**
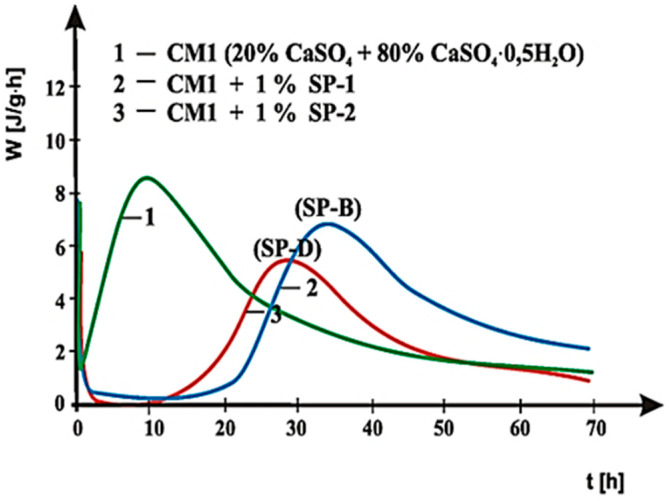
Hydration heat evolution rate curves for CM1 cement without and with SP-1 and SP-2 superplasticizers.

**Figure 26:**
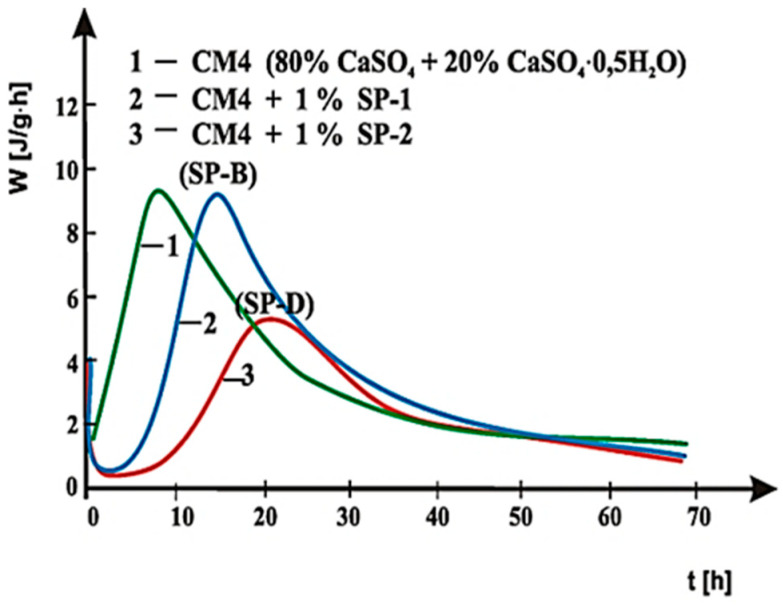
Hydration heat evolution rate curves for CM4 cement without and with SP-1 and SP-2 superplasticizers.

Curves of hydration heat evolution rates for cements C1, C2 and C3 containing various forms of the calcium sulphate, CaSO_4_·0.5H_2_O, CaSO_4_, and CaSO_4_·2H_2_O, respectively, and cements CM1, CM2, CM3 and CM4, containing mixtures of sulphates: CaSO_4_·0.5H_2_O and CaSO_4,_ with, and without 1% by mass of SP-1 and SP-2 superplasticizers are presented in Figure 21, Figure 22, Figure 23, Figure 24, Figure 25 and Figure 26. Table 7 presents the total heat evolved for these cements after 12, 24 and 48 h.

With the use of rheological tests, it was demonstrated that the increased concentration of sulphate ions in the paste of C1 cement (containing CaSO_4_·0.5H_2_O) had the influence on reduction of SP-2 maleic superplasticizer efficiency. In that case the silicate effect occurred earlier (after approx. 21 h of hydration) than in presence of SP-1 acrylic superplasticizer (after approx. 38 h), (Table 8). It is a result of much greater delay of silicates hydration by SP-1 superplasticizer (extension of the induction period to ca. 10 h, Figure 21).

As shown in Figure 21, Figure 22 and Figure 23, the introduction of SP-1 and SP-2 superplasticizers to pastes made of C1, C2 and C3 cements causes a shift of a silicate effect on heat evolution curves towards longer time, and thus a delay of calcium silicates hydration (Table 8). Addition of the setting time regulator in a form of CaSO_4_·0.5H_2_O, with the highest dissolution rate, to C1 cement (Table 3), and thus, with the highest content of SO_4_^2−^ ions in the paste liquid phase, causes the increase of the silicate effect intensity, as well as the increase of the total cement hydration heat evolved, compared to pastes of C2 and C3 cements, containing sulphates with a lower dissolution rate (Figure 21, Figure 22 and Figure 23). It results from a known impact of sulphates on acceleration of C_3_S phase hydration.

A reaction between polycarboxylate groups-COO^−^ that are present in polymer chains and Ca^2+^ ions from the pastes may have the impact on faster hydration of calcium silicates in the paste (at high concentration of sulphate ions) containing SP-2 superplasticizer compared to SP-1 superplasticizer. On one hand it leads to reduced quantity of the active polymer in the solution in result of its building into chelates being formed [53,54]. On the other hand, binding of carboxylate groups into chelate complexes with calcium ions may lead to faster transition of Ca^2+^ ions to solution from C_3_S phase [38,55], which could explain accelerated hydrolysis of silicate phases in presence of SP-2 superplasticizer.

Replacement of the calcium sulphate hemihydrate with sulphates that have lower dissolution rate (CaSO_4_ and CaSO_4_·2H_2_O) causes a reduction of intensity and the delay in time of the silicate effect location on the heat evolution rate curve, depending on the type of the superplasticizer. For C2 cement that contains anhydrite (CaSO_4_) in the presence of SP-1 superplasticizer the silicate effect was observed after ca. 13 h, while for C3 cement with a dihydrate gypsum—after 21 h (Figure 22 and Figure 23).

In the presence of SP-2 superplasticizer, the silicate effect was observed after a lapse of ca. 35 h, whereas for C3 cement after ca. 38 h, with the induction period extended to ca. 15 h (Figure 22 and Figure 23).

The above test results indicate that SP-2 superplasticizer in C2 and C3 cements delays hydration of silicates to a larger extent than SP-1 superplasticizer. It is also confirmed by the results of hydration heat testing for cements CM1, CM2, CM3 and CM4 (Figure 24, Figure 25 and Figure 26, Table 7), containing calcium sulphates (CaSO_4_·0.5H_2_O and CaSO_4_) as the setting time regulator. It is illustrated by Figure 25 and Figure 26, where for comparison, curves of cements hydration heat evolution rates are presented in the presence of SP-1 and SP-2 superplasticizer, with the highest and the lowest content of sulphate ions, respectively. A reduction in sulphate ions concentration in CM4 cement by application of a mixture composed of sulphates containing 80% by mass of CaSO_4_ and 20% of CaSO_4_·0.5H_2_O, in the presence of SP-1 superplasticizer, leads to appearance of the silicate effect already after ca. 13.5 h. Whereas, in the presence of SP-2 superplasticizer, this effect appears only after ca. 22 h of hydration, and its intensity is much reduced (Figure 26). The highest concentration of sulphate ions (20% by mass of CaSO_4_ and 80% of CaSO_4_·0.5H_2_O) leads to appearance of the silicate effect in presence of SP-2 maleic superplasticizer after ca. 28 h, compared to location of that effect in presence of SP-1 acrylic superplasticizer observed only after ca. 34 h, i.e., 6 h later. The induction period in this case is extended to ca. 15 h (Figure 25). A similar relationship was also found for CM2 and CM3 cements, and the induction period in those cements was extended to 5 h (Figure 24) and for CM2 SP2 seems to shift the peak heat time (Location of the silicate effect) to the left compared to SP1 the opposite is evident for CM3. The observed difference in the rate of appearance of the silicate effect, originating from the hydration of silicate phases for cements, CM2 and CM3, in the presence of SP-1 and SP-2 superplasticizers, results from differences in composition of these cements and specifically in the type and amount of sulphates used, as well as sensitivity of the superplasticizer to the presence of SO_4_^2−^ ions. This is related to the different availability of SO_4_^2−^ ions in the cement paste depending on the sulphate dissolution rate (Table 3).

Therefore, the lower concentration of sulphate ions in the paste of CM3 cement compared to CM2 cement is due to the sulphate mixture used in their composition containing respectively: (40% by mass CaSO_4_ and 60% CaSO_4_·0.5H_2_O) in CM2 and (60% by mass CaSO_4_ and 40% CaSO_4_·0.5H_2_O) in CM3 (Figure 24). As can be seen, CM2 cement contains more sulphate hemihydrate (60% CaSO_4_·0.5H_2_O) with a higher dissolution rate, which directly affects the higher availability of sulphate ions in the paste. As shown by rheological and heat of hydration testing, maleic superplasticizer SP-2 shows less effective performance in the presence of increased concentration of sulphate ions (60% CaSO_4_·0.5H_2_O in CM2), other than in the presence of SP-1 acrylic superplasticizer. This is confirmed by the accelerated hydration and setting process of CM2 cement in the presence of SP-2, which is associated with a faster loss of this paste fluidity and an accelerated in time appearance of the silicate effect on the heat evolution rate curve than for CM2 cement paste. On the other hand, the content of CaSO_4_·0.5H_2_O reduced to 40% by mass in CM3 cement introduces a smaller amount of SO_4_^2−^ ions, which in the paste containing SP-2 maleic superplasticizer, leads to its higher fluidity and time-delayed appearance of the maximum silicate effect. In this case, the acrylic superplasticizer is less effective.

The above can be explained by the fact that the silicate effect appears earlier in sulphate ion-rich pastes (containing CaSO_4_·0.5H_2_O) (CM2), which is related to the accelerated hydration of calcium silicates.

The greater delay of silicate hydration in the presence of SP-1 acrylic superplasticizer confirmed its greater performance at increased sulphate ion concentration. Under these conditions, the less efficient maleic superplasticizer delays hydration of calcium silicates to the lesser extent than the acrylic superplasticizer.

The heat of hydration tests of cements in the presence of superplasticizers confirmed a relationship between the delay of the cement hydration process and the superplasticizer performance. Maleic superplasticizer (SP-2), with higher hydrophilicity and performance in pastes with proper regulation of the setting process (with lower concentration of sulphate ions) like in CM3, delays hydration of silicates to a greater extent than acrylic superplasticizer (SP-1), in the presence of an earlier the silicate effect, which appears earlier on the heat evolution rate curve (which indicates accelerated hydration).

The higher performance of SP-2 maleic superplasticizer compared to acrylic SP-1 is related to the presence of higher amount of hydrophilic, carboxyl and polyoxyethylene groups in the flexible comb-like structure in the maleic superplasticizer. The backbone chain, adsorbing on the cement grains through a large number of carboxylate groups (COO^−^), effectively prevents flocculation of cement grains due to the presence of very long hydrophilic polyoxyethylene chains, which leads to increased fluidity of the paste and a time-delayed hydration process of the silicate phases.

However, in the case of acrylic superplasticizer, the very small number of carboxylate groups and the shorter side chains in the strengthened ladder-like structure of this superplasticizer cause a smaller range of action in the cement paste (loss of fluidity and accelerated hydration).

In contrast, the performance of the maleic superplasticizer decreases with increasing sulphate ion concentration in the cement paste. Under these conditions the maleic superplasticizer inhibits formation of the gypsum dihydrate. The lower performance of the maleic superplasticizer at high sulphate ion content is most likely due to the competition of the large amount of SO_4_^2−^ ions in the paste with the COO^−^ carboxylate groups of the superplasticizer for the active centres occupied by positive ions Ca^2+^ on the cement grain surface, preventing their adsorption. Therefore, based on increased sulphate ion concentration, binding of carboxylate groups into chelate complexes with calcium ions is likely. This may lead to a faster Ca^2+^ ions transition to solution from the C_3_S phase, which would explain the accelerated hydrolysis of the silicate phase in the presence of SP-2 maleic superplasticizer.

While, in the case of the acrylic superplasticizer, due to a very small quantity of COO^−^ groups, adsorption may occur with partial negative charges formed in result of polarity of bonds in ester groups of the backbone chain. Shorter polyoxyethylene side chains that connect the backbone chain, increase a distance between cement grains, leading to their deffloculation. In that case, the higher availability of sulphate ions in the paste, to a lesser extent, hinders the formation of the steric block and may explain the higher performance of the acrylic superplasticizer in pastes containing gypsum hemihydrate with the high dissolution rate.

It should be emphasized that tests conducted in this study show that sulphates definitely change performance of polycarboxylate superplasticizers. The high concentration of sulphate ions reduces the performance of the maleic superplasticizer. Under these conditions, the acrylic superplasticizer is more effective and is more suitable for fluidity of pastes with higher SO_4_^2−^ ion content. Therefore, sulphate ions concentration in the paste should be taken into account when the superplasticizer choice is made.

It is emphasized that superplasticizer hydrophilicity, adopted in this paper to assess its performance in cement pastes, allows for better prediction of its behavior in the cement paste (with various composition of a liquid phase), as it takes into account the mechanisms of interaction between individual elements of the superplasticizer structure with the cement paste phases.

As in case of C1, C2 and C3 cements containing the following as the setting time regulator: CaSO_4_·0.5H_2_O, CaSO_4_, and CaSO_4_·2H_2_O, respectively, and in the case of CM1, CM2, CM3 and CM4 containing mixtures of calcium sulphates (hemihydrate and anhydrite), reduced total heat evolved is observed along with the increase of the admixture efficiency in the cement paste (Table 7).

Hydration heat tests of cements, in the presence of superplasticizers, showed a relation between the delay of the hydration process of calcium silicates in the cement and efficiency of the superplasticizer in the cement paste. The maleic superplasticizer (SP-2), with higher efficiency, in pastes containing CaSO_4_ and CaSO_4_·2H_2_O, with lower concentration of sulphate ions, delays hydration of silicates to a greater extent than SP-1 superplasticizer with the lower efficiency. Whereas, in pastes with high concentration of sulphate ions, a reverse effect is observed. In these conditions, less efficient SP-2 superplasticizer delays hydration of calcium silicates to the lesser extent than SP-1 acrylic superplasticizer.

Furthermore, comparing the values of Table 8 it is clear that SP-2 is delaying the silicate effect compared to SP-1 in all cases except C1 and CM1. It is closely related to the discussion presented above on the performance of SP-1 and SP-2 superplasticizers and the type of calcium sulphate used (CaSO_4_, CaSO_4_·2H_2_O, CaSO_4_·0.5H_2_O) with different dissolution rates in C and CM cements, observed in the course of silicate phases hydration during heat of hydration tests of these cements.

Cements C1 and CM1 show the higher content of CaSO_4_·0.5H_2_O-sulphate hemihydrate—with the highest dissolution rate, which translates into increased availability of sulphate ions. The C1 cement contains, respectively: 100% by mass CaSO_4_·0.5H_2_O and CM1 its content amounts to 80% by mass. Therefore, for these cements, in the environment of increased concentration of sulphate ions in the paste, the increase in the total heat evolution and acceleration of the hydration process is observed with simultaneous lower performance of SP-2 maleic superplasticizer demonstrated.

## 5. Discussion

The paper contains the analysis of efficiency of polycarboxylate superplasticizers, ester derivatives of the acrylic acid and maleic anhydride after their esterification with polyoxyethylene glycols (PEG) with various molar mass, in cement pastes.

The chemical structure of superplasticizers used was determined with the use of the gel permeation chromatography (GPC) and infrared absorption spectroscopy (FTIR). It was found that superplasticizers-derivatives of the maleic acid-showed a high molecular weight and were built of polymaleic backbone chains and very long polyoxyethylene side chains.

It was demonstrated that acrylic superplasticizers had lower molar mass, and they were built of polyacrylic backbone chains and a larger amount of shorter polyoxyethylene side chains.

As a measure of superplasticizers efficiency, their hydrophilicity was adopted, determined by means of a hydrophilicity coefficient expressed as a ratio of ether band absorbance A_ET_^1110^ (_STR_CH_2_−O−CH_2ET_) of hydrophilic oxyethylene groups to ester band absorbance A_ES_^1730−40^ (_STR_C=O_ES_) of hydrophobic ester groups O=C−O−C (with constant content of aliphatic groups).

Higher hydrophilicity of maleic superplasticizers compared to acrylic superplasticizers results from a larger content of hydrophilic groups than hydrophobic groups in these superplasticizers, as well as additional increase of hydrophilicity of this superplasticizer in result of carboxylate groups (COO^−^) formation from hydrolysis of anhydrides and maleic acids in the cement paste environment.

Based on rheological testing of cement pastes it was found that along with the increase of hydrophilicity of acrylic and maleic superplasticizers, their efficiency increased in pastes containing calcium sulphates CaSO_4_·2H_2_O and CaSO_4_, which ensured the effective delay of C_3_A hydration. The higher efficiency of the maleic superplasticizer compared to acrylic one is associated with presence of hydrophobic, carboxylate and polyoxyethylene groups in the maleic superplasticizer. The backbone chain of that superplasticizer by being adsorbed on cement grains by a large quantity of carboxylate groups (COO^−^) effectively prevents flocculation of cement grains, due to the very long hydrophilic polyoxyethylene side chains, which leads to higher fluidity of the paste.

In case of the acrylic superplasticizer, a very small amount of carboxylate groups, as well as shorter side chains in the rigid, ladder-shaped structure of that superplasticizer, cause a smaller range of its activity in the cement paste.

It was found that the efficiency of the maleic superplasticizer got lower along with the increase of sulphate ions concentration in the cement paste. In those conditions the maleic superplasticizer inhibited formation of the gypsum dihydrate. Lower efficiency of the maleic superplasticizer with high content of sulphate ions in the cement paste most likely results from competition of SO_4_^2−^ ions with carboxylate groups-COO^−^ of the superplasticizer for active centers occupied by Ca^2+^ ions on the surface of cement grains, which reduces their possible adsorption.

In case of the acrylic superplasticizer, due to a very small quantity of COO^−^ groups, adsorption may occur with partial negative charges formed in result of polarity of bonds in ester groups of the backbone chain. Shorter polyoxyethylene side chains that connect the backbone chain, increase a distance between cement grains, leading to their deflocculation. In that case the high concentration of sulphate ions in the paste, to a lesser extent, hinders the formation of the spherical blockade, and may explain the higher efficiency of the acrylic superplasticizer in pastes containing gypsum hemihydrate with the high dissolution rate.

According to the research conducted by the author, sulphates definitely change efficiency of polycarboxylate superplasticizers. High concentration of sulphate ions lowers maleic superplasticizer efficiency. In such conditions, the acrylic superplasticizer is more effective, therefore, it is better to fluctuate suspensions containing more SO_4_^2−^ ions. Therefore, sulphate ion concentration in the paste should be taken into account when the superplasticizer choice is made.

Hydration heat tests of cements in presence of superplasticizers confirmed a relation between the delay of the cement hydration process and the superplasticizer efficiency. The higher efficiency of the superplasticizer, the higher delay of hydration is. The maleic superplasticizer with higher hydrophilicity and efficiency in pastes with proper setting process regulation by addition of the calcium sulphate (CaSO_4_·2H_2_O, CaSO_4_) delays hydration of silicates to a greater extent than less efficient acrylic superplasticizer (the silicate effect appears on the heat evolution rate curve with a great delay.

In pastes with a larger content of sulphate ions (containing CaSO_4_·0.5H_2_O), the silicate effect appears earlier, which is associated with acceleration of silicates hydration. The greater delay of silicates hydration in the presence of the acrylic superplasticizer showed better efficiency of that superplasticizer found at the higher concentration of sulphate ions. In these condition the less efficient maleic superplasticizer (containing a larger amount of COO^−^ groups) delays hydration of calcium silicates to a lesser extent than the acrylic superplasticizer (SP-1). It is most likely caused by a reaction occurring between non-adsorbed COO^−^carboxylate groups present in the superplasticizer backbone chain and calcium ions Ca^2+^ present in the cement paste. Binding of carboxylate groups into chelate complexes with calcium ions may lead to faster transition of Ca^2+^ ions to solution from C_3_S phase, which could explain accelerated hydrolysis of the silicate phase in presence of the maleic superplasticizer (SP-2).

Based on superplasticizer hydrophilicity determined, as well as rheological testing and hydration heat tests, a relation between the content of hydrophilic groups and efficiency of the superplasticizer was defined:

*S* = x·(OCH_2_CH_2_)_n_, for O=C−O−C _=const_ (1)

where: *S*—superplasticizer efficiency

x—a number of polyoxyethylene (polyether) chains

n—a length of oxyethylene mers

On that basis, it was found that, along with the increase of a number and the length of polyether side chains, at the constant content of ester groups in the superplasticizer, the superplasticizer efficiency increased: SP-1 < SP-2.

In addition it was found that efficiency of the superplasticizer was proportional to its hydrophilicity expressed by the hydrophilicity coefficient, and inversely proportional to viscosity of the paste:

(2) $$S=\frac{A^{1110}/A^{1730}}{\eta_{pl}}$$

where: $A^{1110}/A^{1730}$—hydrophilicity coefficient

${\;\eta }_{\mathrm{pl}}$—viscosity

It shall be emphasized that superplasticizer hydrophilicity adopted in this paper to assess the efficiency in cement pastes is a parameter that allows better prediction of its efficiency in cement pastes due to the content of C_3_A phase and sulphate ions in the cement paste.

Therefore, the knowledge of superplasticizer hydrophilicity may be an essential help while selecting a type of superplasticizer admixture in technology of cement-based composite building materials.

Moreover the maleic acid is a stronger acid (K_1_ = 1.2 × 10^−2^) than the acrylic acid (K = 5053 × 10^−5^), therefore it forms COO^−^ ions easier than the acrylic acid:

CaSO_4_ ⇄ Ca^2+^ + SO_4_^2−^ (3)

Sulphate ions originating from CaSO_4_ dissociation may compete with COO^−^ carboxylate ions present in the superplasticizer backbone chain in the process of electrostatic anchoring on the local positive charges on the surface of cement grains. Therefore, along with the increase of SO_4_^2−^ concentration, the desorption of carboxylate groups from the surface of cement grains may occur or the adsorption of these groups may be hindered. Along with the increase of SO_4_^2−^ concentration, the ionic strength grows, which causes shrinking (curling) of carboxylate and ether chains, and reduction of adsorption of carboxylate groups on the cement grains surface, as well as desorption of SP chains already anchored on the cement surface may occur. Also Ca^2+^ ions, coming from CaSO_4_ dissociation, neutralize COO^−^ carboxylate groups of the superplasticizer causing their deactivation through formation of calcium acrylates or calcium maleates (with a lower dissolution rate and slower dissociation in water than calcium acrylates or sodium maleates). The lower activity of the maleic superplasticizer than the acrylic superplasticizer in presence of high concentration of Ca^2+^ and SO_4_^2−^, may be caused by lower dissolution rate in water of calcium maleates compared to calcium acrylates.

Knowledge of superplasticizer hydrophilicity may provide essential help during the selection of a superplasticizer admixture in cement-based concrete technology.

## 6. Conclusions

Based on analyses of chemical composition and structure of superplasticizers, as well as their efficiency in cement pastes containing different amount of the SO_4_^2−^ ions that depends on the dissolution rate of sulphates used, it was found that:

- A superplasticizer efficiency depends on its molecular structure. The most significant features are: Hydrophilicity, pure polymer content, weight-averaged molecular mass (Mw) of the superplasticizer polymer, presence of free carboxylic acids and anhydrides in solid samples, content of free poly(ethylene glycols) non-reacted with acid and anhydride.
- SP-2 superplasticizer that contains the shorter backbone chain, long side chains and greater number of carboxylate groups (COO^−^) shows higher hydrophilicity than SP-1 superplasticizer that is built of the longer backbone chain with shorter side chains and contains fewer COO^−^ groups.
- It was found that the superplasticizer (SP-2), with the higher hydrophilicity showed the higher efficiency in pastes containing calcium sulphates (gypsum dihydrate and anhydrite) used as the setting time regulator, than the superplasticizer with lower hydrophilicity (SP-1), despite belonging to the same polycarboxylate group.
- SP-1 superplasticizer with lower hydrophilicity and shorter side chains with the long backbone chain that contains small number of COO^−^ group, is more resistant to the impact of sulphates. The increase of SO_4_^2−^ ions content in the paste by the introduction of CaSO_4_·0.5H_2_O or increasing amounts of CaSO_4_·0.5H_2_O in the mixture with CaSO_4_ generally does not change the viscosity of pastes, but it does promote greater delay of silicates hydration.
- In case of SP-2 superplasticizer that shows higher hydrophilicity and has the short backbone chain and more COO^−^ groups (which suggests its better efficiency) the increase of SO_4_^2−^ ions content in the paste by introduction of CaSO_4_·0.5H_2_O or increasing amount of CaSO_4_·0.5H_2_O in the mixture with CaSO_4_, causes deterioration of rheological properties (reduction of fluidity degree) and affects the acceleration of silicates hydration.

## Figures and Tables

**Table 1:** Chemical and mineral composition of clinker (K) and its Blaine specific surface.

Component	SiO_2_	Fe_2_O_3_	Al_2_O_3_	CaO	MgO	SO_3_	Na_2_O	K_2_O	Cl^−^	CaO_free_	C_3_S	C_2_S	C_4_AF	C_3_A	Blaine Surface [m^2^/kg]
Clinker K [wt%]	21.0	3.1	5.3	65.9	0.8	1.4	0.1	0.9	0.04	0.3	66.0	11.0	10.0	8.7	314.0

**Table 2:** Composition of cements for testing.

Cement	Type of Cement Setting Regulator	Clinker	SO_3_ Content [% by Mass]		
		[% by Mass]	CaSO_4_	CaSO_4_·0.5H_2_O	CaSO_4_·2H_2_O
C1	100% CaSO_4_·0.5H_2_O	95.8	-	2.33	-
C2	100% CaSO_4_	96.0	2.33	-	-
C3	100% CaSO_4_·2 H_2_O	95.0	-	-	2.33
CM1	20% CaSO_4_ + 80% CaSO_4_·0.5H_2_O	95.8	0.47	1.86	-
CM2	40% CaSO_4_ + 60% CaSO_4_·0.5H_2_O	95.9	0.93	1.40	-
CM3	60% CaSO_4_ + 40% CaSO_4_·0.5H_2_O	95.9	1.40	0.93	-
CM4	80% CaSO_4_ + 20% CaSO_4_·0.5H_2_O	96.0	1.86	0.47	-

**Table 3:** Concentration of SO_4_^2−^ sulphate ions in water solutions CaSO_4_·0.5H_2_O, CaSO_4_·2H_2_O and CaSO_4_, after a lapse of different dissolution times, [mg/L].

Time [min]	Concentration of SO_4_^2−^ [mg/L]		
	CaSO_4_·0.5H_2_O	CaSO_4_·2H_2_O	CaSO_4_
10	1,544.41	700.53	559.68
20	1,599.62	1078.20	574.91
30	1,777.82	1147.45	639.65
40	1,876.31	1221.47	898.46
60	1,966.06	1268.98	945.60

**Table 4:** Hydrophilicity and distribution of ether groups between SP polymer and free PEGs developed based on [41].

SP Specimen	SP Hydrophilicity	Mass Fraction of Polymer		Absorbance of Ether Band	
	A_ET_^1110^/A_ES_^1730^	in SP (Fraction 1)	in PEG (Fractions 2 and 3)	in SP (Fraction 1)	in PEG (Fractions 2 and 3)
SP-1	3.30	0.517	0.483	0.793	0.742
SP-2	4.53	0.825	0.175	1.195	0.254

**Table 5:** Yield stress τ_0_ [Pa] and plastic viscosity η_pl_ [Pa∙s] values of cement pastes with addition of calcium sulphates in presence of the superplasticizer after 10 and 60 min.

Type of Paste	Time [min]	Without Superplasticizer		1% SP-1	1% SP-2
		τ_0_ [Pa]	η_pl_ [Paּs]	η_pl_ [Paּs]	η_pl_ [Paּs]
C1(clinker+CaSO_4_·0.5H_2_O)	10	69.9	0.63	0.38	0.51
	60	105.9	0.94	1.49	1.50
C2 (clinker + CaSO_4_)	10	74.6	0.36	0.31	0.16
	60	68.2	0.67	0.80	0.74
C3 (clinker + CaSO_4_·2H_2_O)	10	54.9	0.32	0.30	0.10
	60	52.9	0.43	0.49	0.33

**Table 6:** Yield stress τ_0_ [Pa] and plastic viscosity η_p_ [Pa∙s] values of cement pastes with addition of 5% by mass of CaSO_4_·0.5H_2_O and CaSO_4_ in quantity from 20 to 80% by mass in presence of 1% by mass of superplasticizer type: SP-1 and SP-2.

Type of Paste	Time, t[min]	1% SP1	1% SP2
		η_pl_ [Paּs]	η_pl_ [Paּs]
CM 1 (Clinker + 20% CaSO_4_ + 80% CaSO_4_·0.5 H_2_O)	10	0.41	0.36
	60	1.33	1.55
CM 2 (Clinker + 40% CaSO_4_ + 60% CaSO_4_·0.5 H_2_O)	10	0.33	0.30
	60	1.20	1.39
CM 3 (Clinker + 60% CaSO_4_ + 40% CaSO_4_·0.5 H_2_O)	10	0.32	0.29
	60	0.89	1.05
CM 4 (Clinker + 80% CaSO_4_ + 20% CaSO_4_·0.5 H_2_O)	10	0.30	0.20
	60	0.74	0.75

**Table 7:** Total hydration heat evolved for C1, C2, C3, as well as CM1, CM2, CM3 and CM4 cements without and with SP-1 and SP-2 superplasticizers.

Type of Paste	Quantity of Heat Evolved [J/g] after		
	12 h	24 h	48 h
C1	131.7	264.1	383.0
C1 + SP-1	42.7	148.1	317.2
C1 + SP-2	17.3	134.9	201.4
C2	93.7	171.5	251.1
C2 + SP-1	30.7	111.4	242.5
C2 + SP-2	5.2	16.8	159.2
C3	66.5	122.6	160.4
C3 + SP-1	3.1	71.5	138.7
C3 + SP-2	2.6	19.3	81.6
CM1	66.1	133.2	191.5
CM1 + SP-1	1.7	5.0	126.1
CM1+ SP-2	4.2	28.6	125.8
CM2	68.7	137.2	193.4
CM2 + SP-1	10.5	101.4	184.4
CM2 + SP-2	5.9	52.9	160.3
CM3	69.5	138.9	198.6
CM3 + SP-1	30.2	117.9	185.4
CM3 + SP-2	5.5	84.5	180.8
CM4	71.6	140.6	205.1
CM4 + SP-1	23.2	112.7	191.3
CM4 + SP-2	7.2	57.1	122.6

**Table 8:** Location of the silicate effect for pastes containing SP-1 and SP-2 superplasticizers in time [hours].

Type of Cement	SP-1	SP-2
C1	38	21
C2	13	35
C3	21	38
CM1	34	28
CM2	16	18
CM3	14	21
CM4	13.5	22

## Data Availability

Data is contained within the article.
